# Marginal Damage of Methane Emissions: Ozone Impacts on Agriculture

**DOI:** 10.1007/s10640-022-00750-6

**Published:** 2023-02-21

**Authors:** Jon Sampedro, Stephanie Waldhoff, Marcus Sarofim, Rita Van Dingenen

**Affiliations:** 1 Joint Global Change Research Institute, Pacific Northwest National Laboratory, College Park, MD, USA; 2 U.S. Environmental Protection Agency (USEPA, 6207A), 1200 Pennsylvania Ave NW, Washington, DC 20460, USA; 3 European Commission, Joint Research Centre (JRC), Ispra, Italy; 4 Basque Centre For Climate Change (BC3), Leioa, Spain

**Keywords:** Agriculture, Air pollution, Economic damages, Integrated assessment, Methane, Ozone

## Abstract

Methane directly contributes to air pollution, as an ozone precursor, and to climate change, generating physical and economic damages to different systems, namely agriculture, vegetation, energy, human health, or biodiversity. The methane-related damages to climate, measured as the Social Cost of Methane, and to human health have been analyzed by different studies and considered by government rulemaking in the last decades, but the ozone-related damages to crop revenues associated to methane emissions have not been incorporated to policy agenda. Using a combination of the Global Change Analysis Model and the TM5-FASST Scenario Screening Tool, we estimate that global marginal agricultural damages range from ~423 to 556 $2010/t-CH_4_, of which 98 $2010/t-CH_4_ occur in the USA, which is the most affected region due to its role as a major crop producer, followed by China, EU-15, and India. These damages would represent 39–59% of the climate damages and 28–64% of the human health damages associated with methane emissions by previous studies. The marginal damages to crop revenues calculated in this study complement the damages from methane to climate and human health, and provides valuable information to be considered in future cost-benefits analyses.

## 1 Introduction

Methane (CH_4_) is a greenhouse gas (GHG) that directly drives warming, which leads to physical and economic impacts on many sectors, including agriculture, energy, human health, and biodiversity, associated with climate change (Harmsen et al. 2019; Smith et al. 2020). Methane also causes additional damages through driving the process of formation of tropospheric ozone (O_3_), including impacts to human health (Malley et al. 2017; Turner et al. 2016), climate (IPCC 2013), biodiversity (Unger et al. 2020), and crops and vegetation (Ainsworth 2017; Emberson 2020; Emberson et al. 2018). The 12-year lifetime of CH_4_ makes it a relatively short-lived GHG. However, it is longer-lived than most other O_3_ precursors (nitrogen oxides, non-methane volatile organic compounds, and carbon monoxide), whose atmospheric lifetimes are measured in weeks to months (IPCC 2013). In general, reductions in nitrogen oxides and CH_4_ are the most effective actions to reduce O_3_ concentrations (West et al. 2007). In addition, due to the long equilibration time, O_3_ variations driven by changes in CH_4_ emissions are unconstrained by the location of those emissions (Aakre et al. 2018; Van Dingenen et al. 2018a; Wild et al. 2004). Therefore, CH_4_ emission changes in a certain region (e.g. USA) would (with a response time of 12 years) affect O_3_ concentration levels all over the world, which has direct implications for this study. Prior literature has used this relatively uniform response to quantify the O_3_ benefits of CH_4_ emissions reduction anywhere in the world, as in the Social Cost of Methane (SC-CH_4_) (Colbert et al. 2020; Marten et al. 2015; Marten and Newbold 2012; Shindell et al. 2017; Waldhoff et al. 2011) and the marginal health benefits of CH_4_ mitigation (Sarofim et al. 2017). These marginal benefits can be used to conduct cost–benefit analyses, comparing the marginal climate and health benefits of mitigating a tonne of CH_4_ to the cost of that mitigation activity. U.S. Government rulemakings on Oil and Gas (EPA 2020, 2016a) and Landfills (EPA 2016b) have included CH_4_ from the SC-CH_4_ and acknowledged the additional value to reduced mortality. The work presented here adds to this literature by estimating the marginal benefit of CH_4_ mitigation on agricultural systems.

While climate-related factors such as temperature, precipitation, growing season calendars, or carbon dioxide (CO_2_) concentrations have more ambiguous impacts on agricultural yields, depending on the crop and geographic location (Asseng et al. 2015; Calvin and Fisher-Vanden 2017; Snyder et al. 2020), O_3_-related damages are systematically negative for crop growth and productivity (Ainsworth et al. 2012; Fiscus et al. 2005; Fuhrer and Booker 2003; Monks et al. 2015; Shindell et al. 2019). O_3_ has significantly increased since pre-industrial levels (Lamarque et al. 2005; Tarasick et al. 2019), though the location of these increases varies across the globe. In the last 30 years, the shift on precursor emissions from developed economies to developing regions has reduced O_3_ levels in North America and Europe (Cooper et al. 2014; Fleming et al. 2018; Logan et al. 2012), while those levels have substantially increased in East Asia (Chang et al. 2017; Xu et al. 2020; Zhang et al. 2020). These increases result in significant economic losses on agriculture and food security related threats, which will be increasingly important in the coming decades given the challenge of sustainably feeding an increasing global population (Searchinger et al. 2014).

Prior work has quantified yield losses and/or economic damages associated to O_3_ concentration for current and future periods using different methodologies. Exposure-response-function models (ERFs), which calculate the productivity losses for different crops given the O_3_ concentration level, have been an extensively applied method to estimate both relative yield losses and the subsequent economic damages at global and regional levels for current and/or future periods (Avnery et al. 2011a, 2011b; Chuwah et al. 2015; Feng et al. 2019; McGrath et al. 2015; Sampedro et al. 2020; Sharma et al. 2019; Tang et al. 2013; Van Dingenen et al. 2009; Wang and Mauzerall 2004). While the ERF models are a well-accepted methodology to estimate yield impacts, recent studies conclude the amount of O_3_ absorbed by the plant is a more accurate indicator than the exposure in order to estimate relative yield losses (Ronan et al. 2020). Several studies make use of these dose–response or flux-based models, which consider the O_3_ uptake by plants, to estimate yield productivity losses (Grünhage et al. 2012; Mills et al. 2011, 2018a, 2018b; Peng et al. 2019; Pleijel et al. 2019). However, these flux-based models require ancillary data available at high time and spatial resolution, limiting their application in global studies. Even though the methods, parameters and assumptions are substantially different across the type of models and studies, they all find that high O_3_ levels generate significant relative yield and economic losses.

Recent literature has analyzed the O_3_-related yield losses attributable to CH_4_ and other precursor emissions, and conclude that, while the effects of decreasing NO_x_ or CO are more ambiguous and depend on the geographical location, CH_4_ reductions would significantly improve crop productivity in all regions (Avnery et al. 2013; Shindell et al. 2019; Shindell 2016). However, there are no studies that estimate the marginal impact of a tonne of CH_4_ on agricultural systems. Our study fills that gap in literature and estimates the O_3_-related damages to crop revenues associated with CH_4_ emissions at a global scale. Applying an innovative methodology based on the use of both an atmospheric chemistry and an integrated assessment model, we analyze the marginal damages attributable to different CH_4_ pulses in USA under multiple assumptions (e.g. year of the pulse) in order to provide a robust range of marginal damages at both global and regional levels. Our results suggest that the global marginal damages to crop production range from ~ 423 to 556 $2010/t-CH_4_ (hereinafter $/t-CH_4_). These marginal damages complement a previous study (Sarofim et al. 2017), where the authors estimate O_3_-related marginal damages to human health associated to CH_4_ emissions.

## 2 Study design

In this work we estimate O_3_-driven marginal damages to crop revenues for a central scenario, by implementing a shock in CH_4_ emissions in USA. Because the damages will be affected by a range of assumptions, such as the underlying socioeconomic development pathway, the size and the year of the CH_4_ pulse, or the discount rate used, we estimate the impact under multiple sensitivities in order to test the robustness of our results to these variables (Sect. 2.1). The methodology (Sect. 2.2) is based on an integrated modelling framework that connects two different models, namely the Global Change Analysis Model (GCAM) and TM5-Fast Scenario Screening Tool (TM5-FASST). The details of these models can be found in Appendix I. By combining and processing the outputs of these models, we calculate the O_3_-driven marginal damages to crop revenues for each region and crop.

### 2.1 Scenarios

The marginal damages of a tonne of CH_4_ on crop yields are directly affected by several variables. We test the sensitivity of our results to the size of the pulse (*PulseSize*), the year in which the pulse is implemented (*PulseYear*), the future socioeconomic and technological change pathway (*Storyline*), and the discount rate (*DR*) that is used for discounting future damages to current values.

For the *Storyline*, we make use of the Shared Socioeconomic Pathways (SSPs) (O’Neill et al. 2014), which present five different narratives with different demographic and economic assumptions (Dellink et al. 2017; Samir and Lutz 2017); energy-system assumptions on electricity technologies, fuel preferences, or building energy demands; future agricultural yield improvement rates; and food demands. In terms of emissions, each SSP includes sector, fuel, region, and period specific emission factors for air pollutans (non-GHG), as described in Rao et al., (2017). We estimate the damages for three different *PulseSizes*: 5%, 10%, and 15% of the GCAM projected USA CH_4_ emissions in 2020 of ~ 28 Tg, or 1.42 2.84 and 4.25 Tg, respectively. *PulseYear* also has a direct impact on the marginal damages, since each period has different crop production and emissions, so we have considered 4 different time periods where the pulse is implemented, namely 2020, 2030, 2040, and 2050. We also explore the sensitivity of the results to different rates for discounting the future damages, an uncertain parameter that has been extensively discussed in existing literature (Nordhaus 1994; Stern 2006). We use a range of moderate static values (2%, 3%, and 5%) and three additional dynamic discounting rates, based on the GDP per capita growth of each region, so that the discounting changes over time and by region. For calculating these dynamic rates, we apply the Ramsey formula (Ramsey 1928):

(1) $$Dr_{r,t}=\rho +\eta *g_{r,t}$$

where *Dr* is the discount rate in region *r* and period *t*, *ρ* is the pure time preference, *η* is the coefficient of relative risk aversion, and *g* is the growth rate. We test three different time preference parameters (ρ = 0.1%, ρ = 1%, and ρ = 3%) with the relative risk aversion equal for all the regions and periods (η = 1).

Our main results are calculated for a central scenario generated by combining the SSP2 storyline (“Middle of the Road”), a pulse of 2.84 Tg (10%) in 2020 and using a rate of 3% for discounting future damages (see *Discussion*). All the other *PulseSize*-*Storyline*-*Pulse-Year*-*DR* combinations have also been calculated and shown for sensitivity analysis. This is summarized in Table 1. We note that the pulse is based on a share of USA CH_4_ emissions in 2020. A given pulse size is used consistently over each time period to isolate the effects of the alternative sensitivity dimensions, rather than linking the pulse to the corresponding SSP narrative, which would confound the impact of the pulse size and underlying emissions.

### 2.2 Methodology

**Fig. 1:**
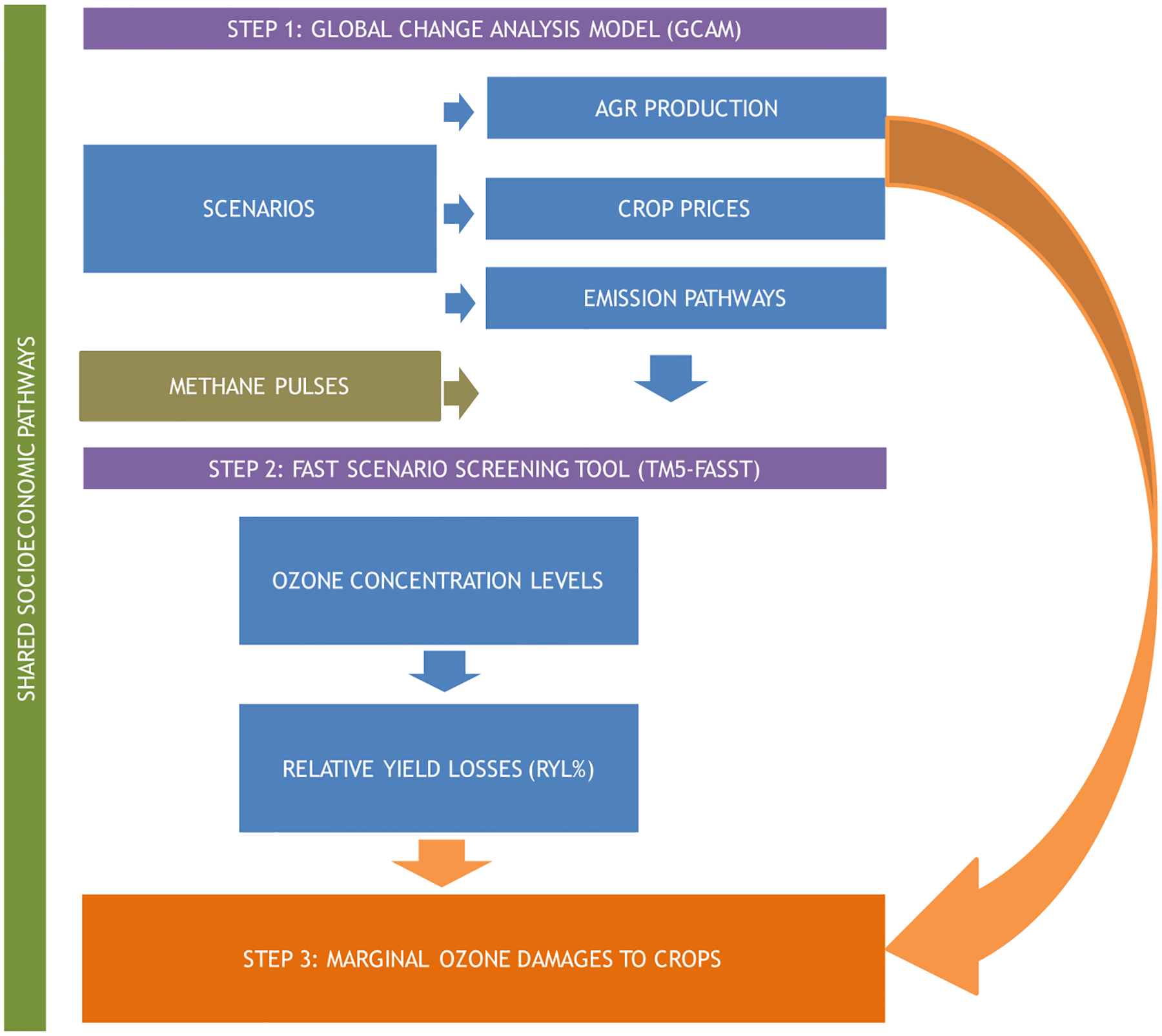
Overview of the methodology

The methodology consists of a sequential connection of two different models (Fig. 1), namely the Global Change Analysis Model (GCAM v5.2), and the TM5-Fast Scenario Screening Tool (TM5-FASST) (Appendix I). We use GCAM to estimate future GHG and air pollutant emissions and agricultural production and prices by region, period, and crop. We note that GCAM projects the agricultural production for all FAO crop commodities classified into twelve aggregate crop categories, as described in online documentation.^1^ The mapping between GCAM crop categories and the FAO commodities is included in Appendix II.

We then translate those GHG and air pollutant emissions to TM5-FASST in order to obtain the O_3_ concentration levels. Given that the half-life of CH_4_ is 12 years (IPCC 2013), the O_3_ formation in a certain year would not only be affected by those year emissions, but also by the CH_4_ emissions from the previous 30 years. Therefore, a CH_4_ shock in a certain period has direct implications in O_3_ formation in subsequent years. In order to capture that dynamic, which is relevant for this study, we transform the O_3_ concentration values obtained from TM5-FASST (“steady-state” concentration) so they follow the actual timeline of formation after a pulse on CH_4_ emission (“transient” approach). This transformation, which improves the calculations with a more realistic emissions-concentration relation (CH_4_–O_3_), is explained in detail in Appendix I.

Using these adapted O_3_ concentration values, we estimate relative yield losses (hereinafter RYLs) using the Weibull exposure–response functions from Wang and Mauzerall (2004). These RYLs are estimated for four different crops (maize, rice, soybeans and wheat) and for the 56 countries/regions in TM5-FASST. Therefore, these damages need to be re-scaled to GCAM regions and expanded to the rest of the crop categories. The RYLs from the 56 TM5-FASST regions are downscaled to country level and re-aggregated to GCAM regions (see Appendix III) based on 2010 data on harvested area from Geophysical Fluid Dynamics Laboratory.^2^ RYLs calculated for four crops have been extended to the rest of the GCAM categories based on their carbon fixation pathway. Significant differences between C3 and C4 plant species in terms of stomatal conductance or transpiration rates will directly affect their sensitivity to damages attributable to O_3_ exposure (Knapp 1993; Leisner and Ainsworth 2012). Therefore, maize RYL coefficients are applied to C4 crop types, while a weighted average of wheat and rice (or wheat, rice and soybeans, in some cases^3^) RYL coefficients are applied to C3 categorized commodities. This procedure ensures that the damages calculated in this study cover all the agricultural production.

We have also estimated RYLs for two symmetric scenarios within each SSP narrative for all different pulses, one that includes the CH_4_ pulse (*PulseScen*), and other that does not incorporate it (*noPulseScen*). So, by difference we are able to estimate the additional RYLs directly attributable to a determined pulse for each scenario (*n*), period (*t*), region (*i*) and crop (*j*) with the following equation:

(2) $$\text{Pulse}.RYL_{n,t,i,j}=\Delta RYL_{n,t,i,j}=RYL_{n,t,i,j}\left(\text{PulseScen}\right)-RYL_{n,t,i,j}\left(\text{noPulseScen}\right)$$

Once having estimated the crop damage coefficients directly driven by the CH_4_ shock, economic damages to crop revenues for a determined scenario (*n*), period (*t*), region (*i*) and crop (*j*) are calculated as:

(3) $$\text{Damag}e_{n,t,i,j}={\text{Prod}}_{n,t,i,j}*{\text{Price}}_{n,t,i,j}*\text{Pulse}.RYL_{n,t,i,j}$$

These economic damages are linearly interpolated in order to have year-by-year results. Then, they are aggregated to cumulative values using different discount rates. Note that for cumulative results we consider 50 years after the *Pulseyear*, which is the maximum allowed by GCAM.^4^ Cumulative damages using a determined discount rate (*r*), can be defined as:

(4) $$\text{Damag}e_{n,i,j}=\sum_{t=\text{PulseYear}}^{t+50}\frac{\text{Damag}e_{n,t,i,j}}{r^{\left(t-\text{PulseYear}\right)}}$$

The final step is to transform cumulative damages into marginal damages, by dividing the damage by the CH_4_ pulse. Therefore, the total marginal damages for all crops (*j*), in region (*i*), under the socioeconomic narrative (*n*) would be defined as:

(5) $$MD_{n,i}=\sum_{j}\frac{\sum_{t=\text{PulseYear}}^{t+50}\frac{\text{Damag}e_{n,t,i,j}}{r^{\left(t-\text{PulseYear}\right)}}}{\text{Pulse}}$$

## 3 Results

We focus our discussion of the results on the central scenario, which is defined by a 10% pulse (2.84 Tg) of 2020 CH_4_ emissions in USA in 2020 (*PulseYear*), under the SSP2 socioeconomic storyline (“Middle of the Road”), with a 3% discount rate. The following figures show the projected CH_4_ emissions and the effects of the CH_4_ shock in the central scenario in 2020 in terms of O_3_ concentration, measured by the seasonal 7-h mean daytime O_3_ concentration^5^ (M7) and RYLs for four different crops.

**Fig. 2:**
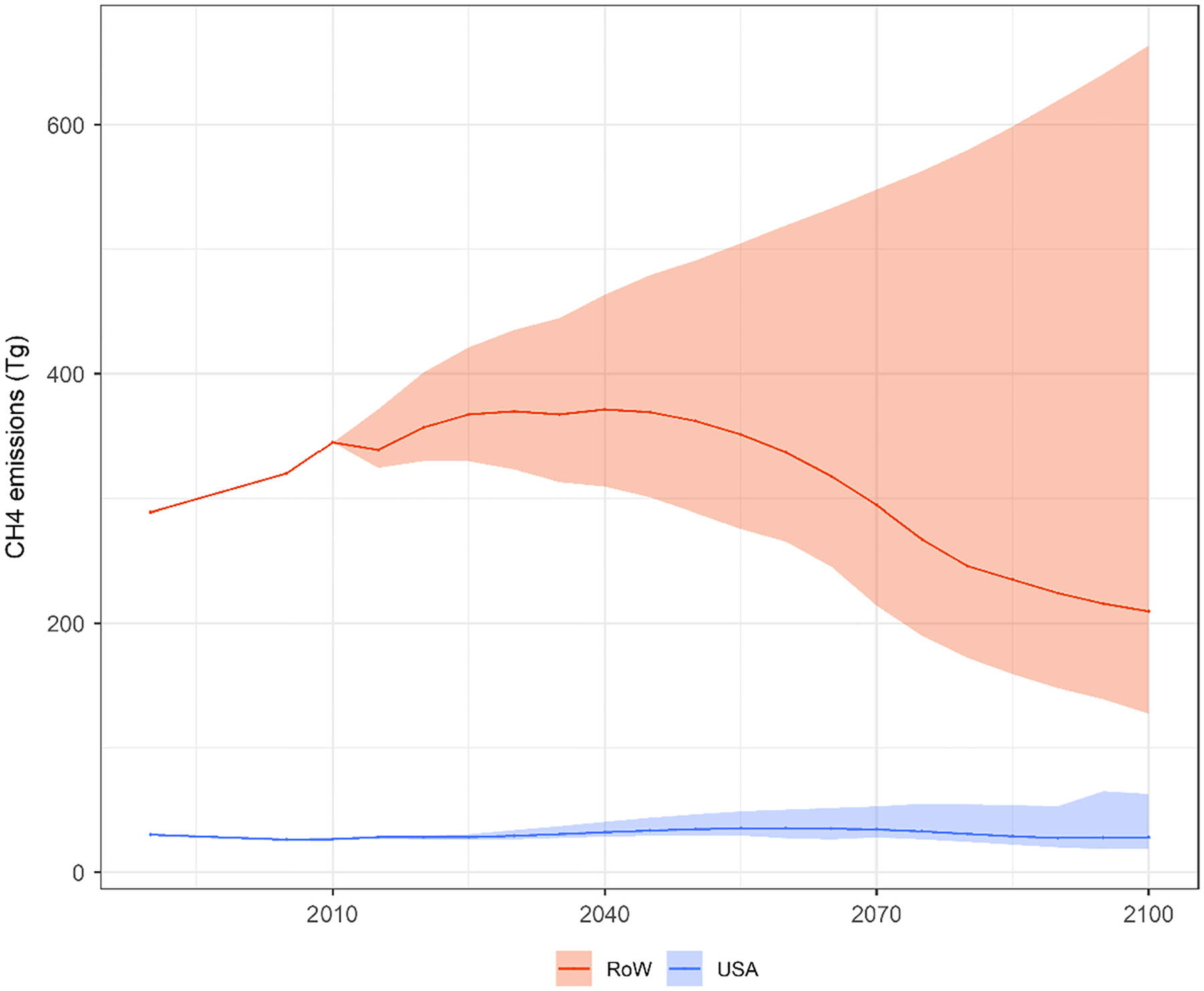
Methane (CH_4_) emissions per period for USA and the rest of the world (RoW) (Tg). The line represents emissions for the SSP2 storyline (Shared Socioeconomic Pathway), while shaded areas represent the ranges determined by all SSP storylines

Future CH_4_ emissions would be substantially different across SSP narratives, as shown in Fig. 2. The differences in CH_4_ emissions driven by SSP storylines are large both at USA and global levels. Globally, SSP1 limits the lower bound while SSP3 limits the upper bound of the CH_4_ emissions trajectory. In the SSP3 narrative, there is a large increase in meat consumption by the end of the century (+ 80%), driven by demand in developing economies, so there is a subsequent increment in the CH_4_ emissions associated with beef, dairy and sheep production. In addition, in the SSP3 narrative there is a large increase on CH_4_ emissions associated to wastewater treatment (Daelman et al. 2012), and the largest increase on CH_4_ emissions from the energy system across the narratives. In the SSP1, the increment on meat demand and the reliance on fossil fuels (associated with energy-system emissions) are the smallest across the narratives, so it projects the lowest CH_4_ levels. Globally, in 2030, compared to SSP2, emissions diverge from − 12% to + 16%. This range increases to (− 20%, + 32%) in 2050, and to (− 38%, + 193%) in 2100. In USA, lower and upper bounds are defined by SSP1 and SSP5, respectively, which have the smallest and largest increments in meat demand, respectively. Taking SSP2 as the point of comparison, uncertainty ranges in USA account for (− 10%, + 26%) in 2030, (− 23%, + 42%) in 2050 and (− 33%, + 125%) in 2100.

**Fig. 3:**
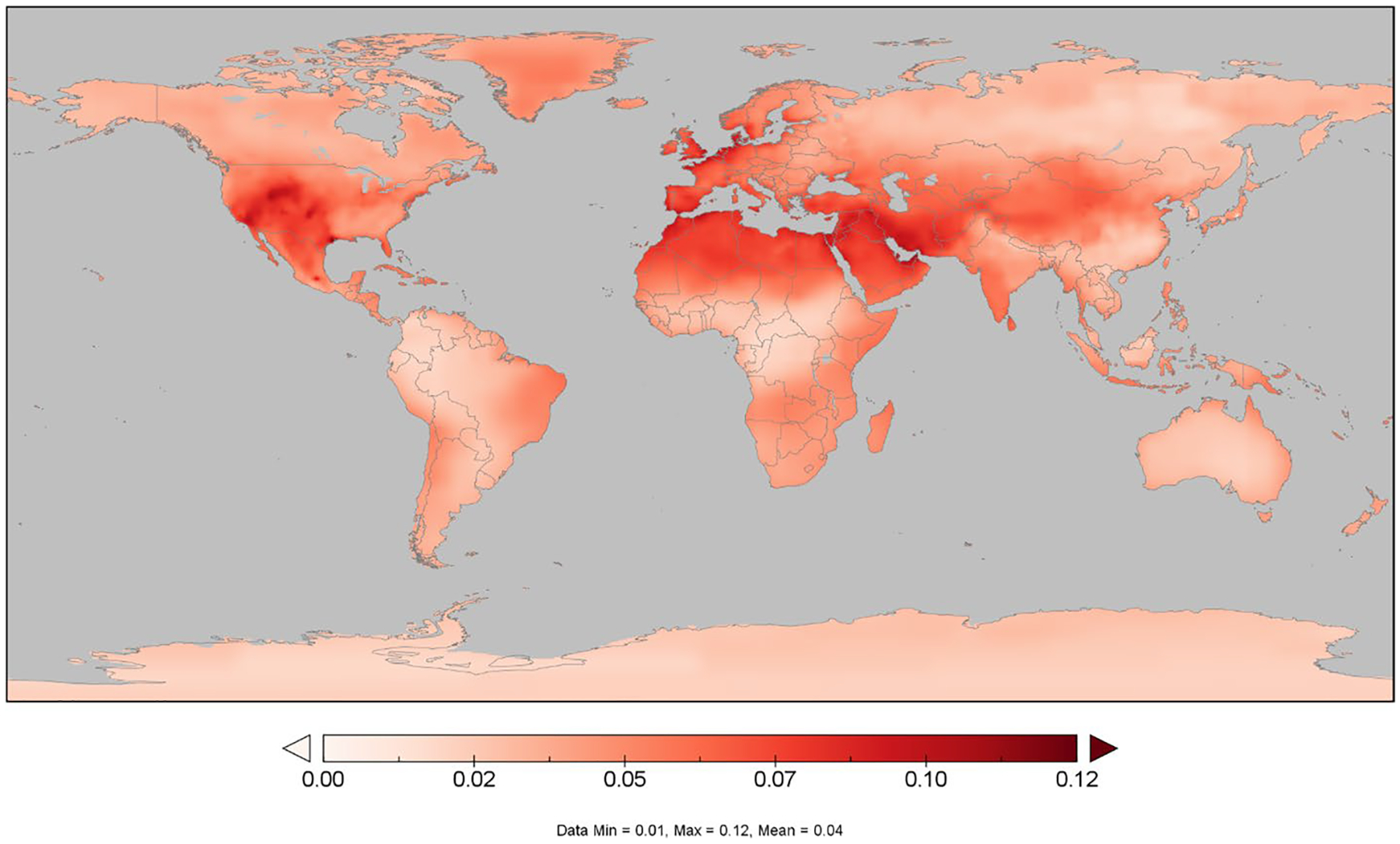
Seasonal 3-monthly mean of 7-h daytime ozone (M7) attributable to the methane (CH_4_) pulse in the central scenario in 2020 (ppbv). We use July as the representative month for this metric. The pulse in the central scenario represents a 10% increase of projected 2020 USA CH_4_ emissions in 2020 under the SSP2 (Shared Socioeconomic Pathway) storyline

**Fig. 4:**
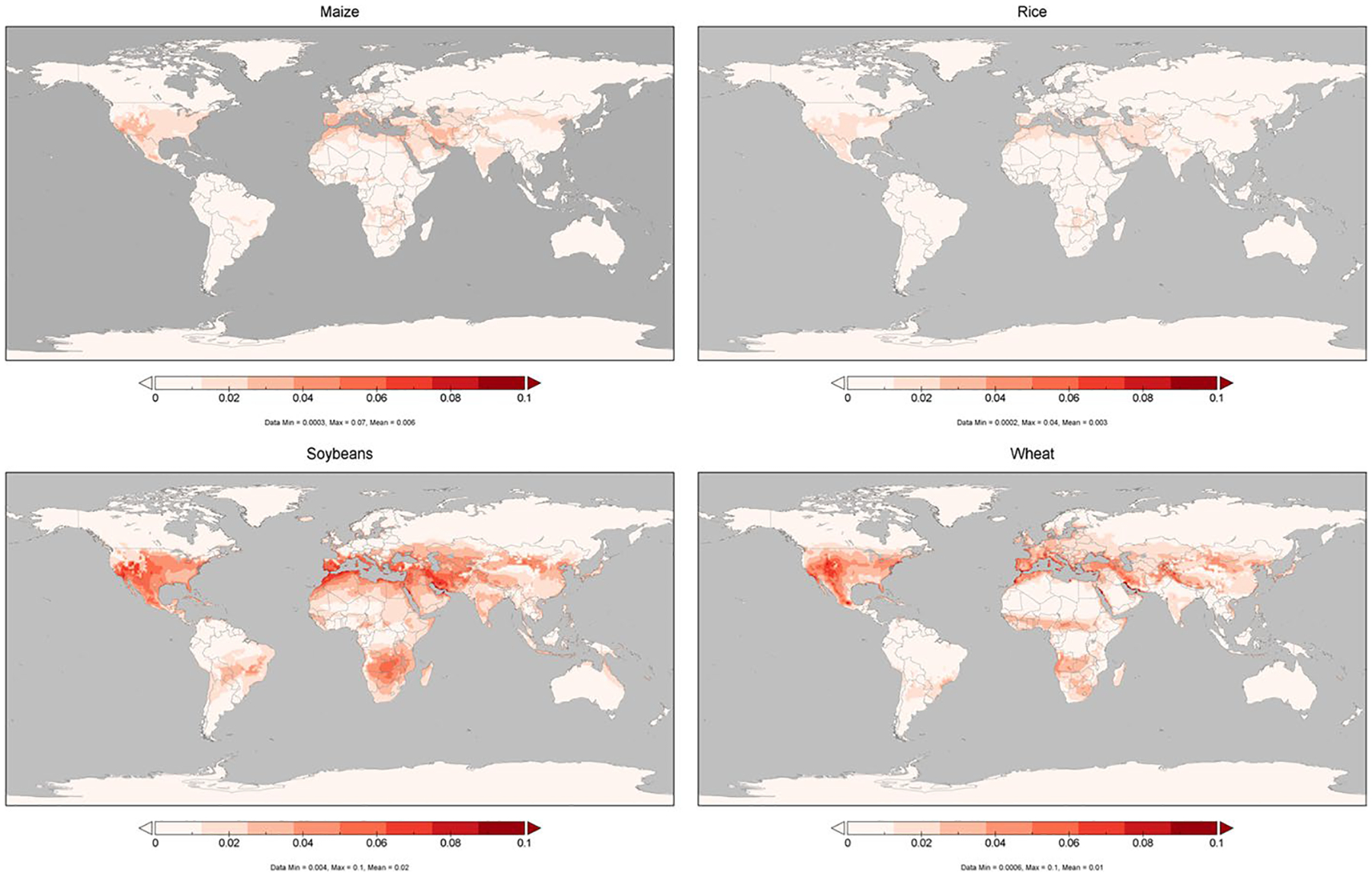
Differences in relative yield losses (RYLs) attributable to the methane (CH_4_) pulse in the central scenario in 2020 for maize, rice, soybeans and wheat (%). The pulse in the central scenario represents a 10% increase of projected 2020 USA CH_4_ emissions in 2020 under the SSP2 (Shared Socioeconomic Pathway) storyline

Figure 3 shows that the CH_4_ pulse would increase O_3_ concentration levels, measured by the 3-month mean of 7-h daytime O_3_ during crop season, up to 0.12 ppbv around the world. Two essential factors for O_3_ formation are emissions of precursor gases and their reaction with solar radiation. Figure 4 indicates that the largest variations in O_3_ levels are located in regions closer to the equator belt, with the highest solar irradiance. We note there are some seasonal differences in O_3_ concentrations, largely associated with NO_x_ emissions. In some regions such as Europe or North America, the large NO_x_ levels could reduce O_3_ concentration through titration at night and during wintertime (Jhun et al. 2015), while this negative relationship plays a minor role during daytime and summertime. Given that, in this study, the O_3_ exposure is calculated to analyze the RYLs, Fig. 3 shows the changes in O_3_ for summertime (July), as it is a representative month for the growing season in those areas with largest crop production (Northern Hemisphere^6^). The RYLs associated to these variations on the growing-season mean of 7-h daytime O_3_ are shown in following figure.

Figure 4 shows that larger RYLs would occur in those regions that suffer larger O_3_ increments, accounting for up to 0.1% in some countries for some crops in 2020. Additionally, the figure demonstrates that there are significant divergences between the crops analyzed, driven by the maximum stomatal conductance which a species can reach. Globally, variations on rice and maize yield damages account for less than 0.02%, while RYL coefficients of soybeans and, to a lesser extent, wheat would increase up to 0.08–0.1% in several regions, such as the Mediterranean area, Middle East, or the West Coast of USA.

**Fig. 5:**
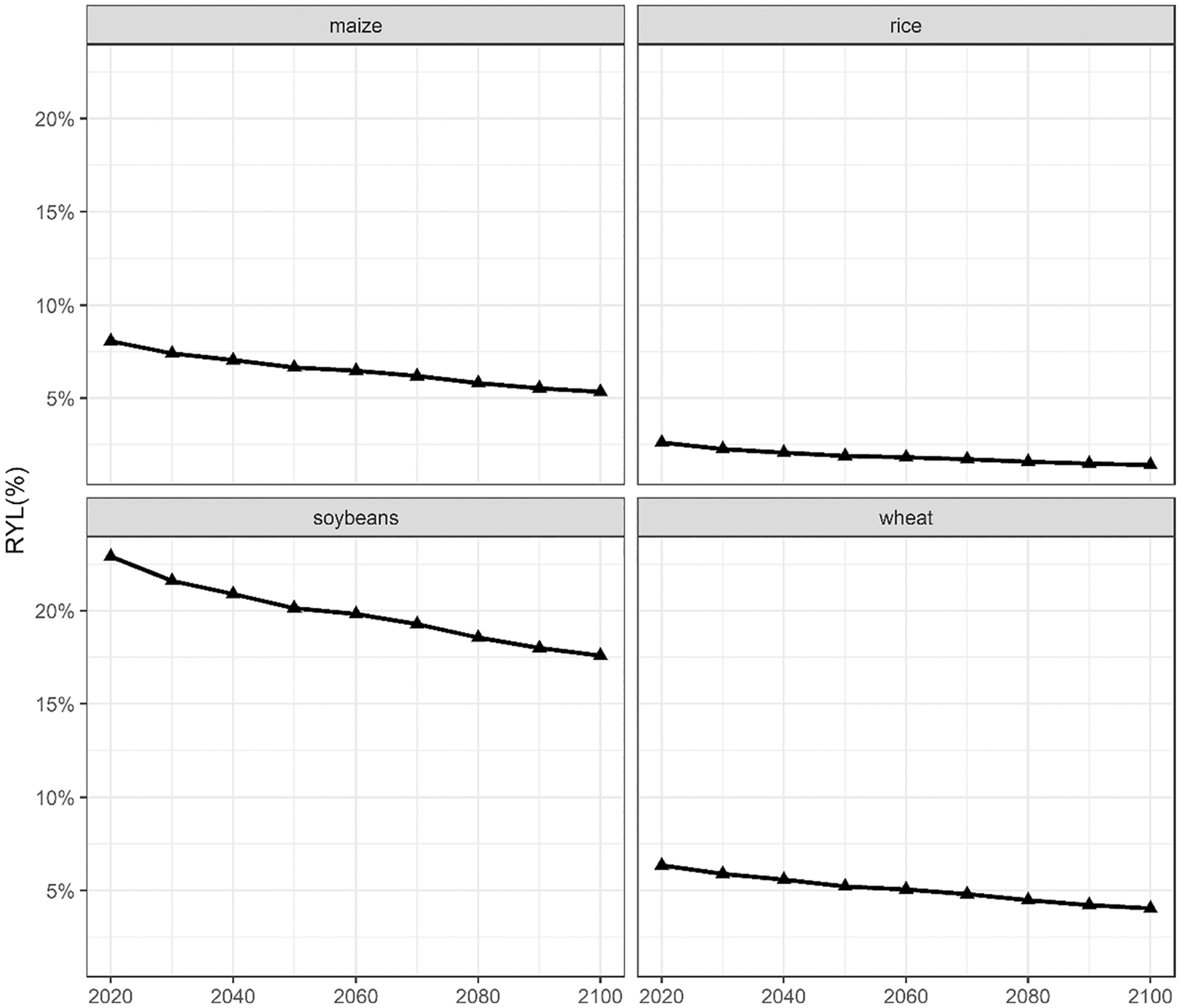
Timetrends of total relative yield losses (RYLs, %) in USA for maize, rice, soybeans and wheat, following the SSP2 (Shared Socioeconomic Pathway) socioeconomic narrative

Focusing on USA, the additional RYLs driven by the CH_4_ pulse in 2020 for the central scenario could represent up to 0.038%, depending on the period and the commodity, with additional impacts in the subsequent periods (see Appendix V). In order to compare the additional RYLs attributable to the central methane pulse with the total RYLs associated to O_3_ exposure in USA, Fig. 5 shows that total RYLs account for 5.3–8.1%, 1.4–2.6%, 17.6–23.0%, and 4.0–6.3% for maize, rice, soybeans, and wheat, respectively during the time horizon analyzed.

**Fig. 6:**
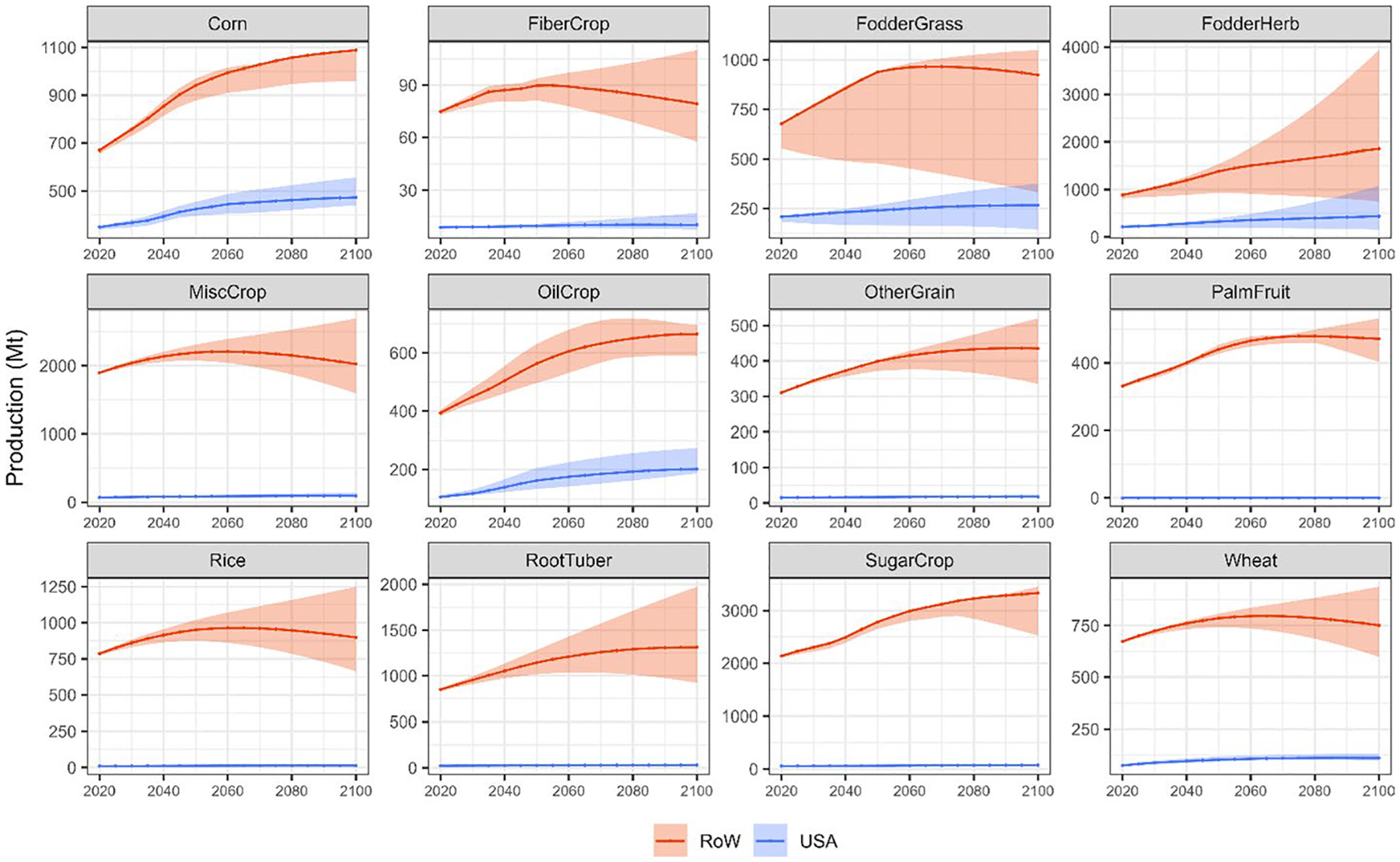
Agricultural production of different commodities per period and region (USA and RoW) (Mt). Lines represent the projections under the SSP2 storyline (Shared Socioeconomic Pathway). Shaded areas represent ranges determined by SSP storylines

**Fig. 7:**
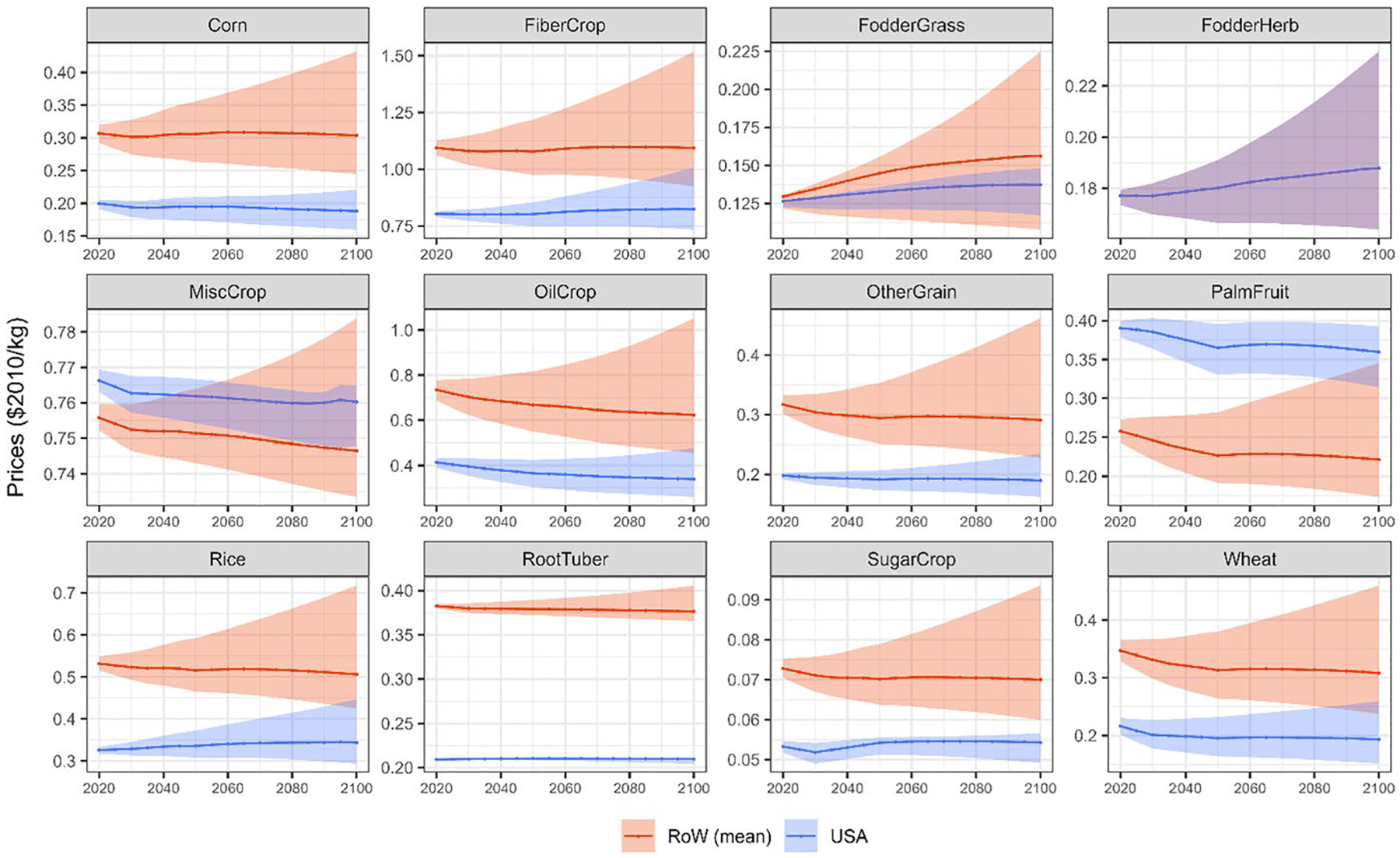
Regional prices of different commodities per period and region (USA and RoW) ($2010/kg). Lines represent the projections under the SSP2 storyline (Shared Socioeconomic Pathway). Shaded areas represent uncertainty ranges determined by SSP storylines. Note that FodderHerb is not regionally traded in the model, so there is a single global price

Once having estimated the additional RYLs driven by shocks in CH_4_ emissions in USA for four representative crops, these are re-scaled to GCAM regions and extended to the rest of the commodities to ensure that the analysis covers all the agricultural production (see Sect. 2.2). Then, multiplying these RYLs by the agricultural production and price of each crop, in each region and period and for each narrative, we can obtain the O_3_-related economic damages to crop revenues driven by CH_4_ pulses (see Methodology). Agricultural production levels and prices in each year are obtained from GCAM and presented in Figs. 6 and 7.

Figure 6 shows that future agricultural production levels vary with the socioeconomic narrative, indicated by the shaded area in the graphs, as population growth rates and food demand parameters are some of the most relevant drivers for crop production. In the SSP2 storyline, agricultural production increases at a decreasing rate up to 2060, where it stabilizes or decreases for some commodities (e.g. MiscCrops). This trend is similar to the global population projection under the SSP2 socioeconomic storyline.^7^ However, some specific crops such as corn, sugar, and oilcrop (the category that includes soybeans) present a continuously increasing production that does not stabilize with population peak. This is largely driven by the non-food (bioenergy) demand for these commodities at a global level. The increases in production in 2100, compared to 2020 values, are 53.4% (40.8–60.5%), 73.4% (59.7–87.5%), and 55.1% (19.7–60.4%) for corn, oilcrop, and sugar, respectively. USA presents similar patterns in terms of future agricultural production levels. In 2020, it is the largest producer of corn and oilcrop, representing 34.2% and 21.2% of global production, respectively. The increasing demand for these two commodities for bioenergy purposes entails a continuous increase of production, which does not stabilize with population peak, similar to the global trend. Therefore, regional production in 2100, compared to 2020, for corn and oilcrop would increase around 35.8% (30.2–57.8%) and 91.0% (81.6–143.9%). In terms of price trajectories, they are also directly related to the socioeconomic narratives. In the SSP2 storyline, price variations are relatively small, as demonstrated in Fig. 7. At a global level, the largest increments in prices in 2100, compared to 2020 levels, are represented by FodderGrass, which increases around 21% followed by FodderHerb (6%). On the other hand, oilcrop (− 15%), palmfruit (− 13%) and wheat (− 11%) represent the largest relative reductions. In USA, FodderGrass (9%) and FodderHerb (6%) also present the largest price increases, closely followed by rice (5%). On the contrary, oilcrop (− 18%) and wheat (− 11%) show the main price relative reductions.

Given that economic damages to crop revenues driven by CH_4_ pulses are calculated by multiplying additional RYLs attributable to the shocks by the agricultural production and price of each crop, marginal damages are calculated by aggregating and discounting those damages and dividing them by the emission shocks, as described in the Methodology section. However, other analyzed variables such as the pulse size (*PulseSize*), the *PulseYear*, the socioeconomic narrative (SSP), or the discount rate have direct impacts on the results. Therefore, we have developed a sensitivity analysis, in order to examine which variables would most impact to marginal damages.

**Fig. 8:**
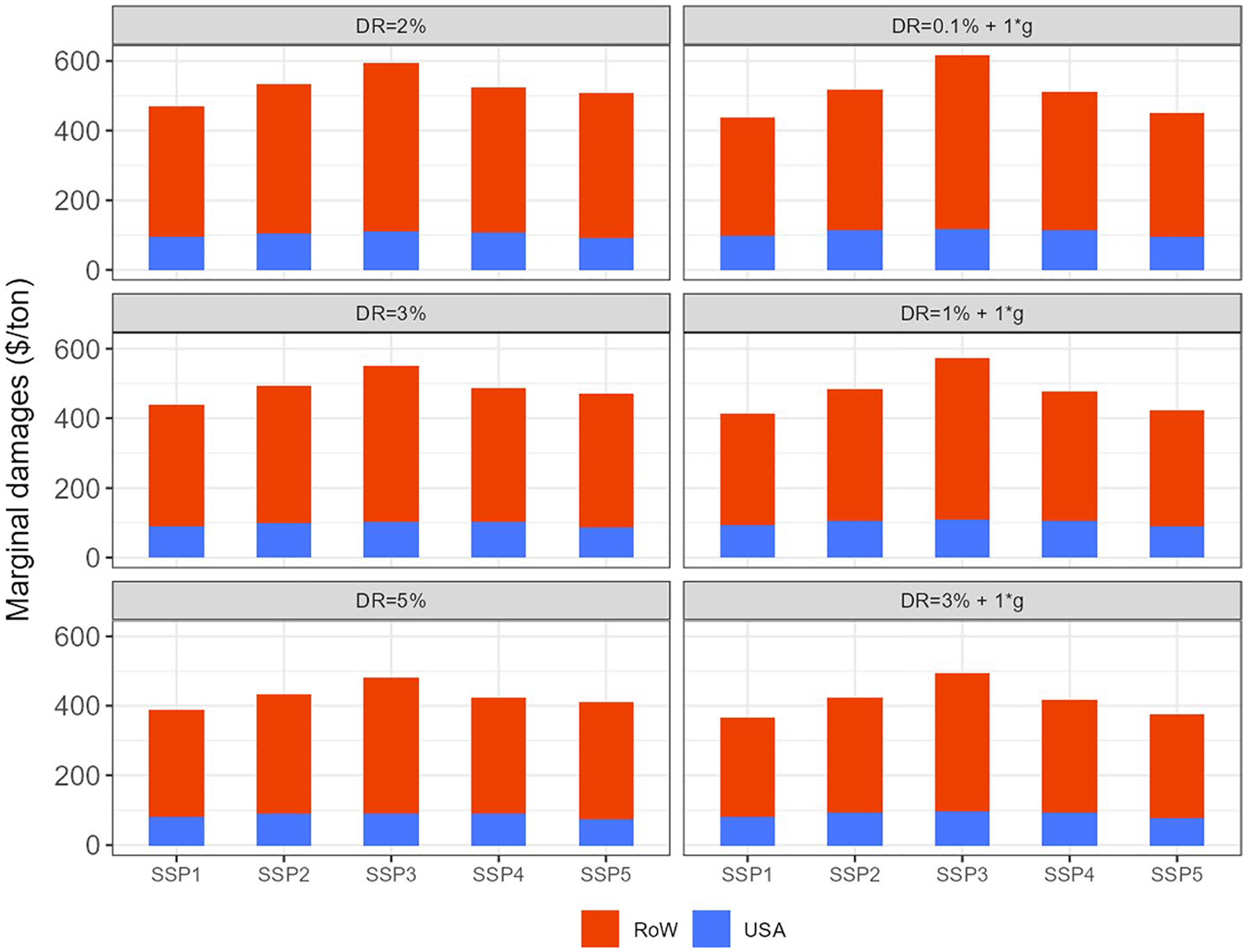
Ozone-related marginal damages to crop revenues driven by a methane (CH_4_) pulse by region, discount rate and SSP storyline (Shared Socioeconomic Pathway) for the central pulse ($2010/t-CH_4_). This pulse represents 10% increase of projected 2020 USA methane emissions in 2020. Damages are calculated, accumulated and discounted for 50 years (*PulseYear* + 50)

Figure 8 shows that, in the central scenario, total marginal damage accounts for 493 $/t-CH_4_, of which 98 $/t-CH_4_ are attributable to damages in USA and 395 $/t-CH_4_ in the rest of the world. The figure shows that the damages wil be affected by different factors such as the socioeconomic narrative (SSP) or the discount rate. *PulseSizes* and *PulseYears* also have a direct impact on O_3_-related marginal damages to crop revenues and they are analyzed in the following figures.

**Fig. 9:**
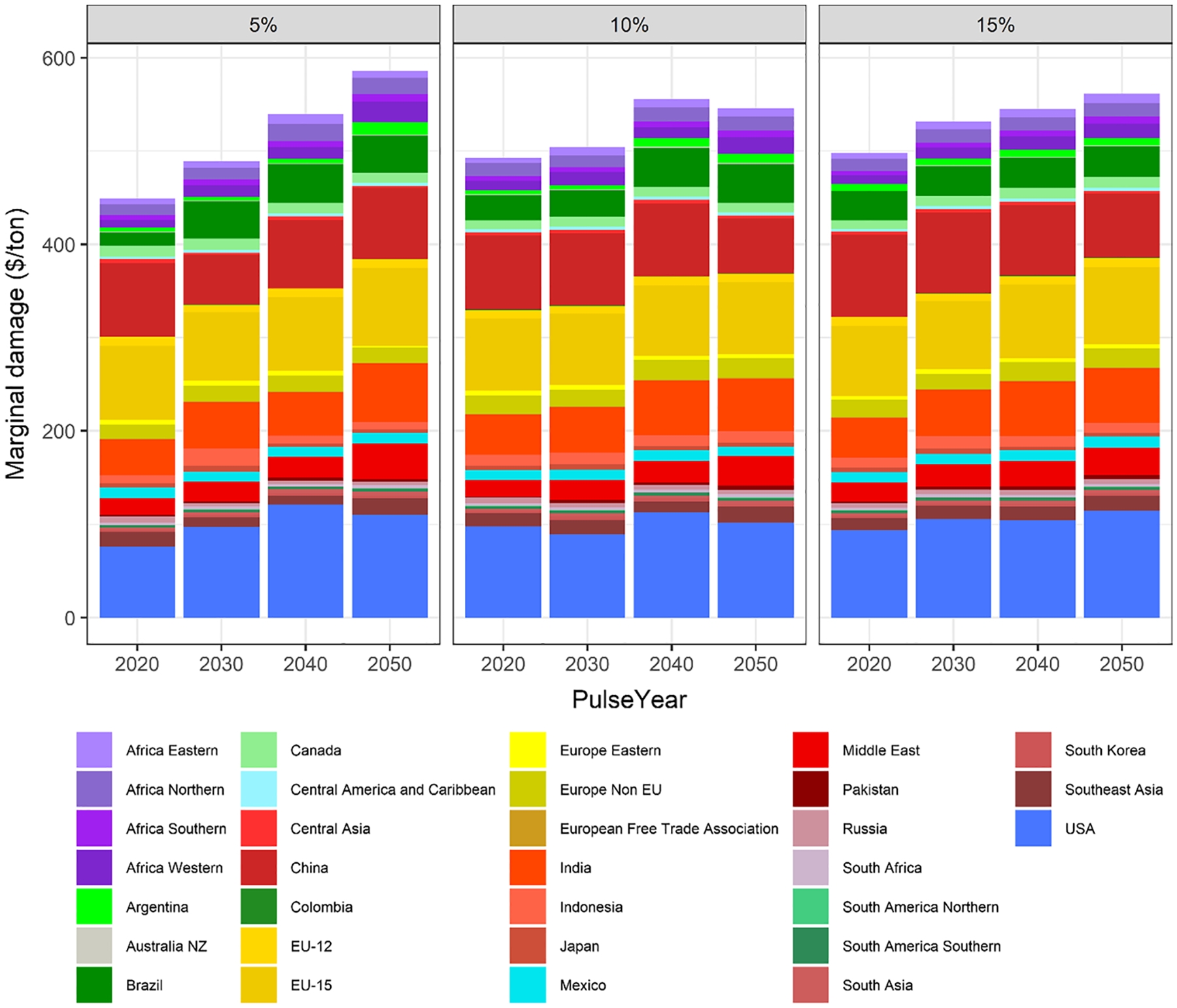
Ozone-related marginal damages to crop revenues driven by a methane (CH_4_) pulse by region, *PulseSize* and *PulseYear* under the SSP2 storyline (Shared Socioeconomic Pathway) and using a discount rate of 3% ($2010/t-CH_4_). Damages are calculated, accumulated and discounted for 50 years (PulseYear + 50). The definition of the GCAM regions is detailed in Appendix III

Figure 9 shows global marginal damage to crop revenues attributable to variations in CH_4_ emissions, broken down for different regions around the world. The most affected regions are USA, China, India, EU-15 and Brazil which are the main crop producers^8^ and, except for the latter, also generate the larger O_3_ precursor emissions during the time horizon analyzed. The Appendix VI includes a table with the marginal damages for each region in the central scenario. Figure 9 also shows that marginal damages do not always increase with increments in *PulseSizes* or time periods (*PulseYears*), due to different factors such as the non-linearity of the exposure–response functions or the implicit threshold in the estimation of the seasonal mean daytime O_3_ concentration (see Methodology). The evolution of some air pollutants, such as NO_x_, will impact these trends: in those regions where these pollutants increase more and the reduction is delayed (e.g. developing Asia), marginal damages tend to increase along with the pulse implementation year. This trend is not observed in those regions where the projected reduction in air pollutants starts in the nearer term (e.g. USA). When the pulse is 5% (1.42 Tg), marginal damages in USA increase until 2040 and they drop in 2050. However, the increases in marginal damages in other regions such as Central Asia or India are the drivers of increasing global values during the analyzed time horizon. Likewise, if the pulse is 15% of the emissions (4.25 Tg), the global marginal damages increase over time. Moreover, in this case USA also presents larger marginal damages in all the periods (including from 2040 to 2050), as the size of the pulse entails additional RYLs increases in every time step. However, the figure shows a different pattern for the 10% pulse (2.84 Tg). At a global level, marginal damages increase up to 2040, when they peak. In 2050, additional RYLs driven by the pulse decrease compared to previous period in some regions that bear most of the marginal damages, namely USA, China or India.

**Fig. 10:**
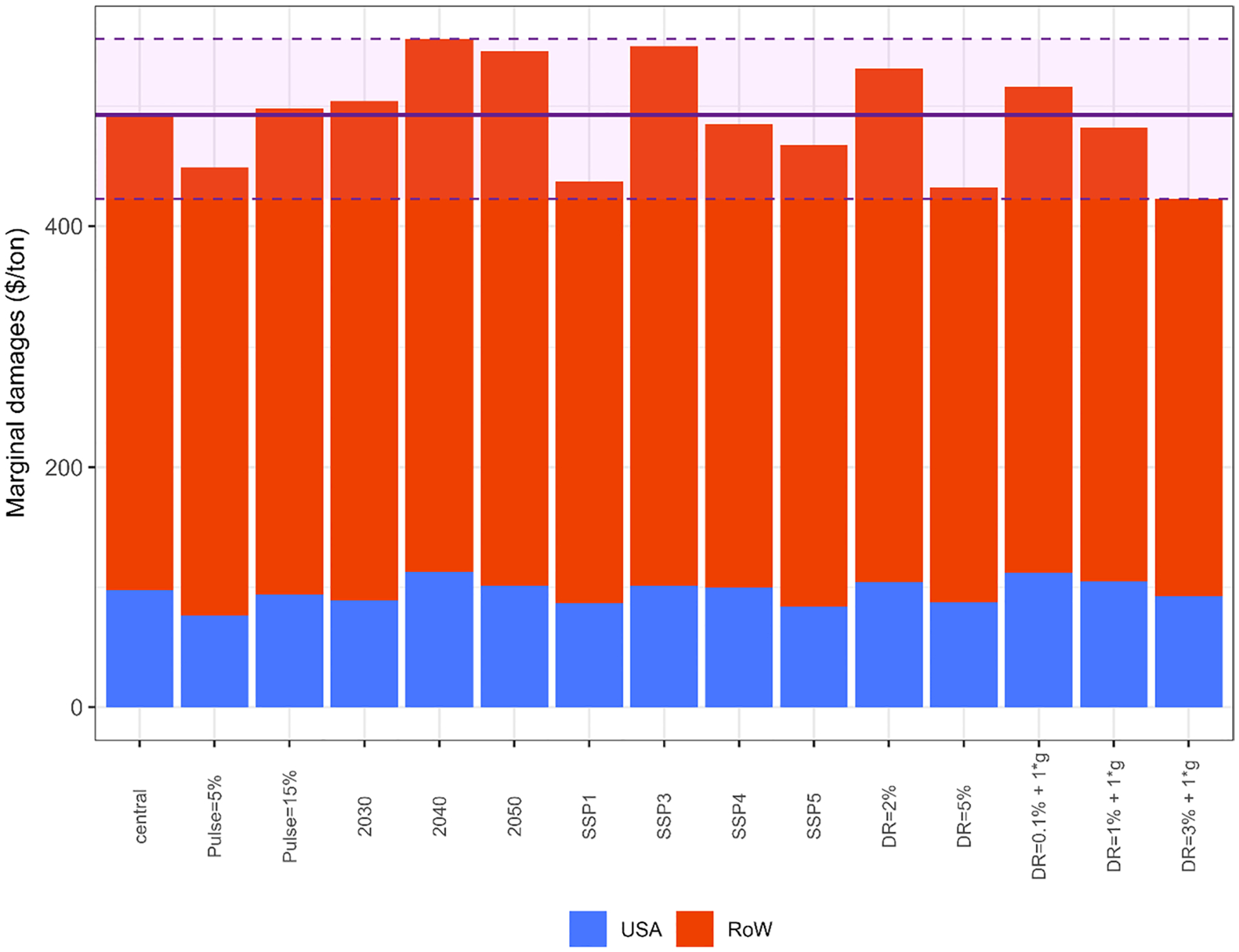
Sensitivity analysis of the ozone-related marginal damages to crop revenues. The central scenario represents a 10% increase of projected 2020 USA methane emissions in 2020 under the SSP2 storyline (Shared Socioeconomic Pathway) and using a discount rate of 3%. The solid line indicates the marginal damages in the central scenario. The dashed lines show the lower and upper bounds, respectively

Finally, Fig. 10 shows the O_3_ related marginal damages to crop revenues for the central scenario and how they vary by modifying each variable individually with respect to central assumptions. In the central scenario marginal damages account for 493 $/t-CH_4_, distributed as 98$/t-CH_4_ in USA and 395 $/t-CH_4_ in the rest of the world. Regarding the uncertainty range, using a higher discount rate would determine the lower bound of the total marginal damages (423 $/t-CH_4_) while setting the *PulseYear* to 2040 would determine the upper bound of the damages (556 $/t-CH_4_). Focusing on USA, the lower *PulseSize* (5%) and setting the pulse to 2040 would indicate the lower and upper bounds of the uncertainty range of the marginal damages, which account for 76 $/t-CH_4_ and 113 $/t-CH_4_, respectively.

## 4 Discussion and conclusion

The release of methane (CH_4_) due to human activities leads to physical impacts and related economic damages in many sectors, including agriculture, energy, human health, vegetation, and biodiversity. The physical impacts arise through two key routes, climate change-through driving warming- and air pollution, as methane is a precursor for the formation of ozone^9^ (O_3_). Improving the understanding of these physical impacts and economic damages is important for policymaking. Cost–Benefit Analysis (CBA) is a prominent tool that is used to assist policymaking in considering damages and the benefits of mitigation to reduce these. CBA requires monetisation of damages, including both the climate and ozone-related impacts of methane releases. To assist the process of monetising methane damages, for consideration in CBA studies, this study focusses on the air pollution damages related to ozone formation, specifically in the form of the economic costs that are produced for agriculture due to crop-yield losses. We estimate that total global crop damages to crop revenues per tonne of methane account for $493 2010$/t-CH_4_ in our central scenario. In order to check the sensitivity of our results to different variables, we re-calculate these marginal damages modifying key parameters, namely socioeconomic narratives, discount rates, pulse sizes, and years when the pulse is implemented. We find that the effect of altering each of these variables is relatively small, ranging from about − 14% to + 13% of our central estimate (about $423 per t-CH_4_ to about $556). Implementing a larger pulse size has only a very modest increase in damages, while a smaller pulse decreases damages by about $44 per tonne. As for the socioeconomic narrative, we find that the SSPs with higher reference yield improvement rates (e.g. SSP1) have smaller damages. The temporal pathway is not completely smooth, as the damages depend on the underlying O_3_ concentrations (which vary across regions over time), where the production is, how much is produced, and what the prices are. Results also show that applying a higher discount rate would have a significant impact as it determines the lower bound of the total marginal damages, demonstrating that the valuation of near-term versus distant damages plays a significant role on the presented results. In terms of regional distribution of these damages, about 19% of the damages occur in USA, with the EU-15 region, China, and India being the next most heavily impacted because they are the largest crop producers and the largest emitters of O_3_ precursors.

The results obtained in this work can complement other estimates of damages from methane. At a global level, the calculated marginal damages to crop revenues under central scenario assumptions are equal to about 39–59% of the climate damages, as estimated by the Social Cost of Methane in different studies (Colbert et al. 2020; Marten et al. 2015; Marten and Newbold 2012; Shindell et al. 2017; Waldhoff et al. 2011). Moreover, the damages estimated in this study would represent 28–64% of the damages associated to increased premature mortality reported in Sarofim et al., (2017). While that study found only about 11% of the global mortality damages are in USA, we find that a large share of the agricultural damages occur in this region (~ 19%), due to its role as a major agricultural producer. Appendix VII includes a comparison between these climate and non-climate (ozone-related) damages.

The study has some caveats and limitations. Damages have been calculated in absence of climate change related impacts. Future changes on temperature and precipitation pathways will also have an impact for crop yields, as reflected in different studies (Lobell et al. 2014; Rosenzweig et al. 2014; Snyder et al. 2021, 2020; Waldhoff et al. 2020). The damages associated with climate change cannot be linearly added up with the ozone-related damages calculated in this study (Da et al. 2021). The aggregation of these different impacts and the effects on global and regional agricultural systems is therefore a complex undertaking that is planned to be explored in future work.

Furthermore, we also acknowledge the uncertainty associated with the rate used to discount future damages to present values. In the central case of the analysis, we apply a discount rate of 3%, because it has been the central value used historically by the U.S. federal Government for rulemaking, following the guidelines of the Interagency Working Group on the SCC (IWG) (IWG 2010; Stock and Greenstone 2021). However, we recognize that the real interest rate on the 10-year Treasury note, which has been used to calibrate the social rate of time preference, has been substantially below 3% for the last couple of decades (Carleton and Greenstone 2021; Council of Economic Advisers 2017). In this line, recent reviews show that lower discount rates (or even declining rates, see Arrow et al. 2013) are now being applied in different regions, including USA (OECD 2018; O’Mahony 2021). In our analysis, we use additional lower and higher, static and dynamic, and region-specific discount rates to capture this uncertainty (see Sect. 2.1), considering its direct implications for the estimation of the monetized damages and relevance for non-substitutable capital. The alternative discount rates we apply in our sensitivity analysis range from 1.5 to ~ 5%, in order to represent the discount rates recommended by the National Academies SCC assessment (National Academies of Sciences 2017), and the most recent scientific literature. For example, a recent report focused on the estimation the social cost of carbon (Rennert et al. 2022) proposed four alternative discount rates of 1.5%, 2%, 2.5%, and 3%. Similarly, Drupp et al. 2018, based on a survey of over 200 experts, recommend to use a median discount rate of 2.3%. All these values have been incorporated to our sensitivity analysis, while declining discount rates will be explored in future research.

In addition, the model used to estimate the O_3_ concentrations and the subsequent yield losses (TM5-FASST) adds the individual O_3_ responses from each precursor as an approximation to obtain the response to combined changes in precursors. While this is clearer for some precursors (e.g. NMVOC), the relation between NO_x_ and O_3_ is more complex with more non-linearities (Wu et al. 2009). However, the O_3_ exposure metrics used in this study, namely the growing-season mean of 7-h (or 12-h) daytime O_3_, is constrained to the daytime and to summer season when the effect of the NO_x_ titration is reduced in most parts of the world. In addition, we use this exposure metric because it has been proven to be more robust than other threshold-based measures (e.g. AOT40). The TM5-FASST documentation paper (Van Dingenen et al. 2018b) includes a detailed validation section where the authors discuss the model performance and the tradeoffs between accuracy and applicability. In addition, a previous study discusses some limitations of the combined used of the models applied in this study, while showing the advantages and disadvantages of using the global exposure–response functions, in contrast to using regional or national data, or using the more detailed “flux-based” models (Sampedro et al. 2020). We also acknowledge that in this study we only calculate the damages to agricultural crops, and we do not include the damages to forest products, pasture, or other ecosystems, which have been proven to be significant in several studies (Emberson 2020; Fuhrer et al. 2016, 1994; Grulke and Heath 2020; Unger et al. 2020; Wittig et al. 2009).

## Figures and Tables

**Table 1:** Summary of the scenarios and assumptions

Variable	Definition	Central assumption	Description	Alternative assumptions
Storyline	Underlying socioeconomic narrative	Shared Socioeconomic Pathway-SSP2	Moderate population and GDP growth, and EFs projections (“Middle of the Road”)	SSP1 SSP3 SSP4 SSP5
PulseSize	Size of the pulse	2.84 Tg	10% of the projected USA CH4 emissions in 2020)	2030 2040 2050
PulseYear	Year when the shock on CH4 emissions is implemented	2020	-	0.001386
DR	Discount rate	3%	Moderate assumption consistent with prior work	2% 5% Ramsey: ρ=0.1%, η=1 Ramsey: ρ=1%, η=1 Ramsey: ρ=3%, η=1

**Fig. 11:**
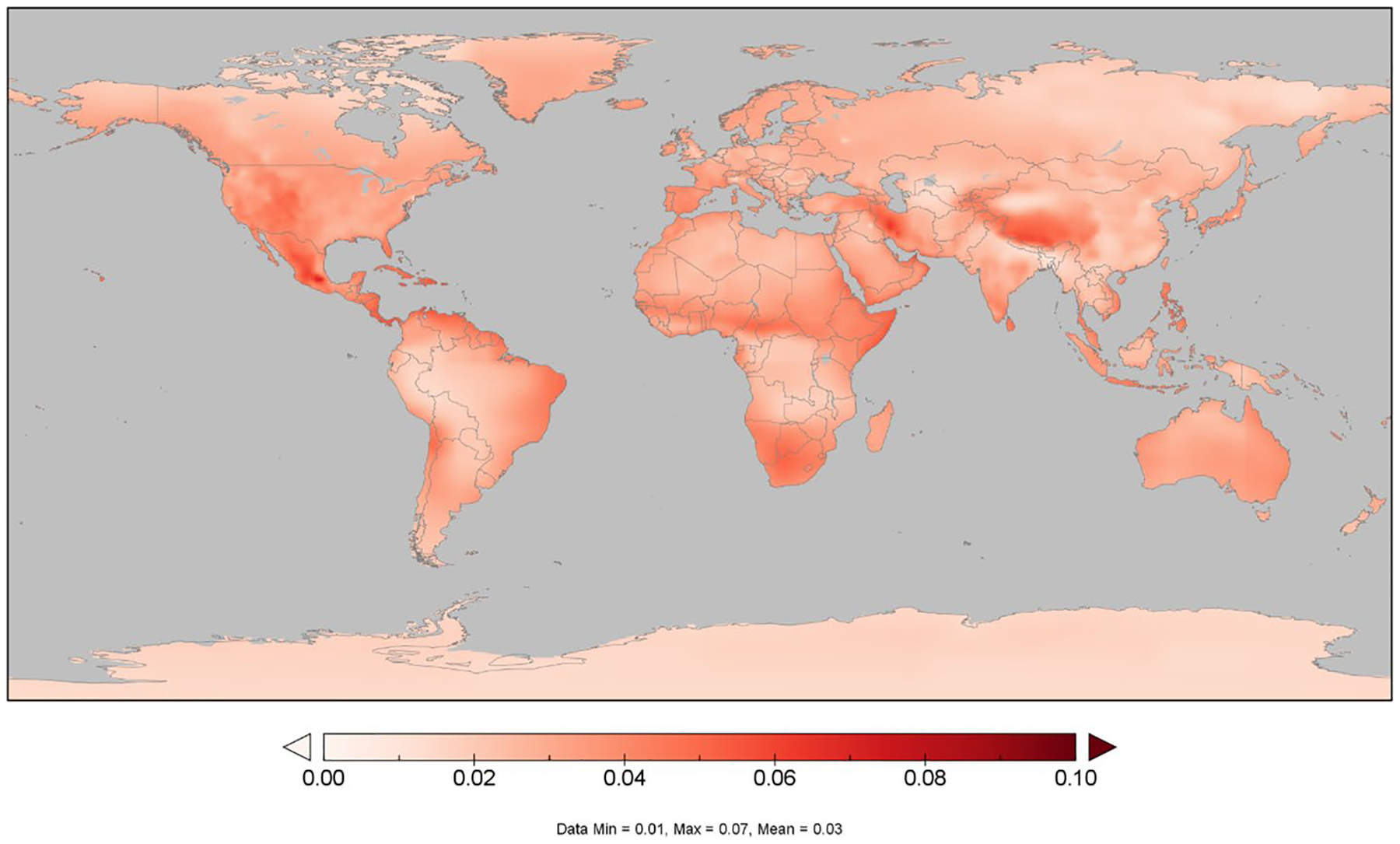
Differences in the seasonal 3-monthly mean of 7-h daytime ozone (M7) attributable to the methane (CH_4_) pulse in the central scenario in 2020 in January (ppbv). The pulse in the central scenario represents a 10% increase of projected 2020 USA CH_4_ emissions in 2020 under the SSP2 (Shared Socioeconomic Pathway) storyline

**Fig. 12:**
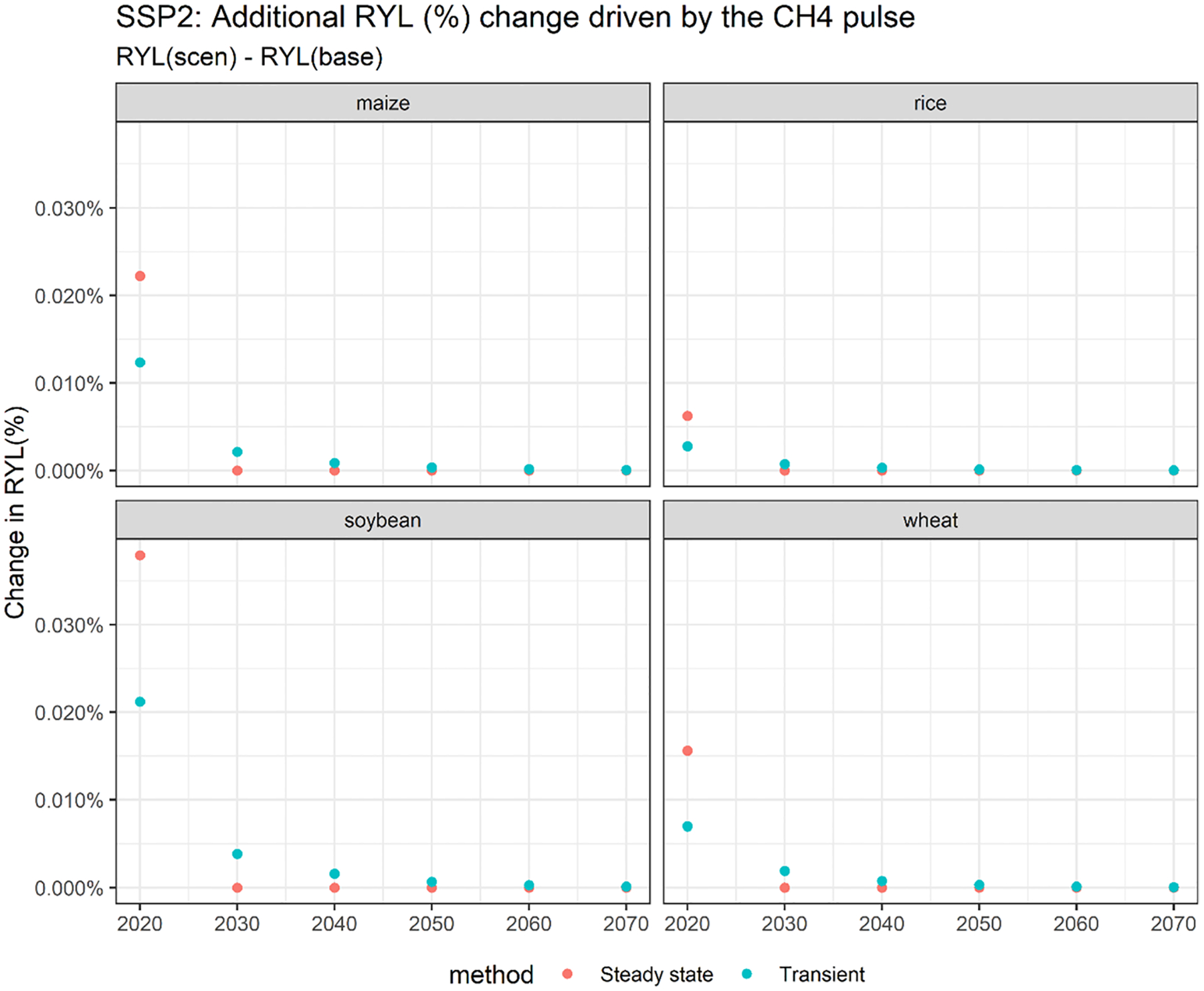
Variation in relative yield losses (RYLs) in USA related to the methane (CH_4_) pulse in the central scenario for maize, rice, soybeans and wheat (%). The pulse in the central scenario represents a 10% increase of projected 2020 USA CH_4_ emissions in 2020 under the SSP2 storyline (Shared Socioeconomic Pathway)

**Fig. 13:**
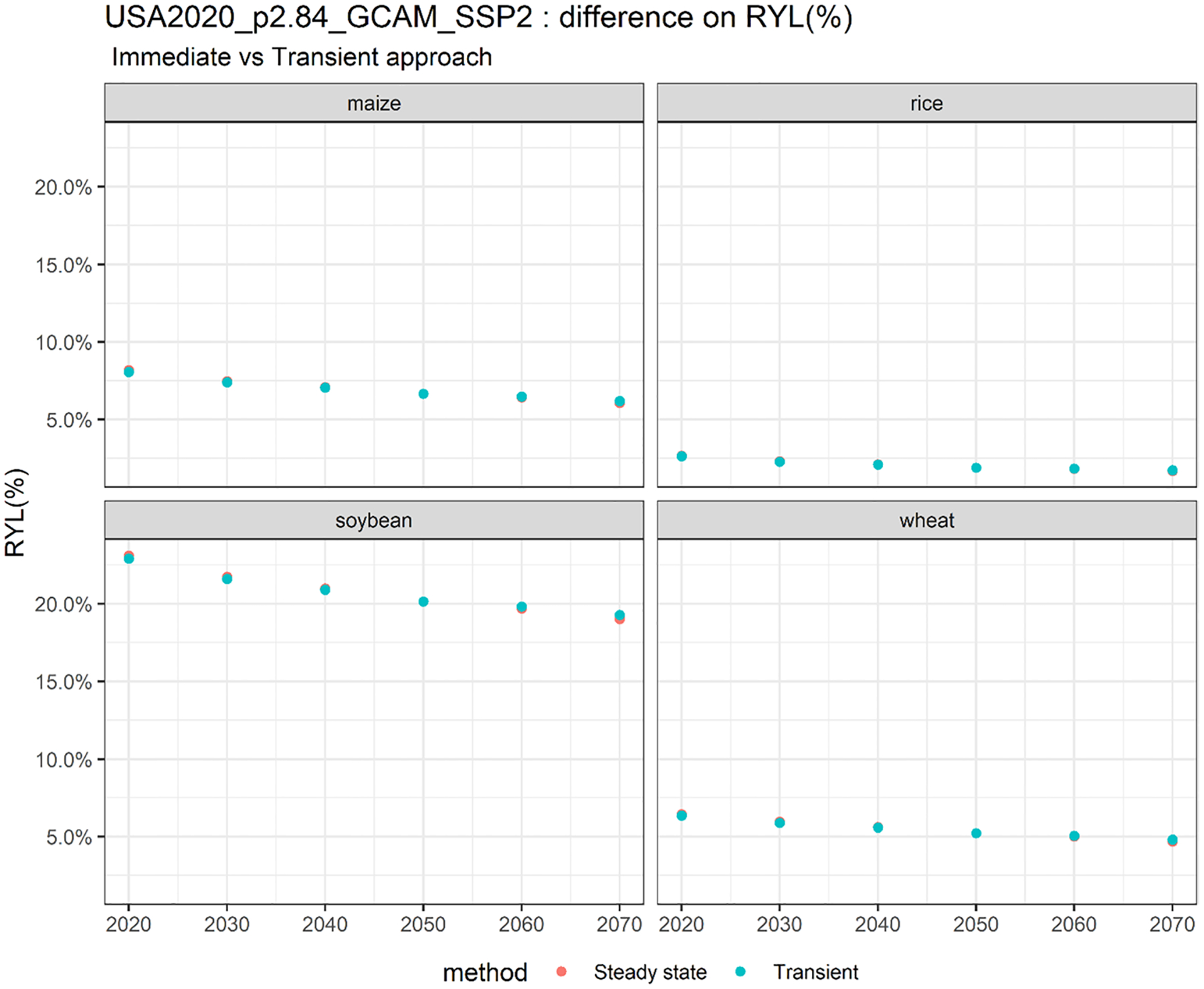
Relative yield losses (RYLs) in USA in the central scenario for maize, rice, soybeans and wheat using the transient and the steady-state approaches (%). The central scenario represents a 10% increase of projected 2020 USA CH_4_ emissions in 2020 under the SSP2 storyline (Shared Socioeconomic Pathway)

## Footnotes

1
https://github.com/JGCRI/gcam-doc/blob/gh-pages/details_land.md

2
https://www.gfdl.noaa.gov/model-development

3 We include soybeans in the weighted averages when legumes are included in the crop category.

4 In this study we analyze the effect of methane pulses in four different “*PulseYears*” which are 2020, 2030, 2040, and 2050. GCAM only simulates up to 2100, so in order to maintain symmetry among the damages, we consider 50 years after the pulse, which is the maximum capability for the *PulseYear* = 2050.

5 We indistinctly consider M7 and M12 as one indicator type (see Appendix I).

6 The Appendix IV shows the variation in January, as a representative month for the growing season in the Southern Hemisphere. Figure 11

7 This storyline (SSP2) assumes that population would reach the peak in 2070 with around 9.5 billion people.

8 In 2020, USA would be the largest producer of corn and oilcrop, representing 34% and 21% of total production, respectively. Wheat would be mainly produced in China (17%) and EU-15 (14%), which is also the main OtherGrain producer (21%). China is also the main MiscCrop producer (37%.). The production of rice is concentrated in three regions, namely China, India and Southeast Asia, that jointly produce around 68% of world rice. Likewise, PalmFruit production is concentrated in Indonesia (40%) and Southeast Asia (41%). The main SugarCrop producer is Brazil (35%), followed by India (17%).

9 Changes in CH4 emissions also have an indirect impact on particulate matter (PM2.5) by an alteration of atmospheric oxidant concentrations, affecting production of sulfates (Anenberg et al. 2012).

## Appendix I: Model Description

### Global Change Analysis Model

The Global Change Analysis Model (GCAM) is an Integrated Assessment (IA) model developed at the Joint Global Change Research Institute. It is a deterministic partial-equilibrium model which represents the interaction of different modules, namely energy, land-use, emissions, water, and climate. GCAM documentation is available online.^10^ In this study GCAM v5.2 version is used. This version builds form GCAM v5.1 (Calvin et al. 2019), with the addition of water supply, water markets, and the regional differentiation of agricultural commodity markets.

In terms of agriculture and land use, the model divides the world in 384 subregions, generated from the combination of geo-political regions (32) with water basins (235). Within each land region, GCAM models 11 types of land: cropland, bioenergy, pasture (managed and unmanaged), forest (managed and unmanaged), shrubland, urban, desert, and tundra, the last three of which are exogenous. In terms of crops, GCAM models around 20 types of crops, with different irrigation and management characteristics that directly affect to yields. Land allocation is based on economic decisions, which follow a logit model based on the relative profitability of using land for competing purposes (McFadden 1973). Each competing land has an average profit over its entire distribution, instead of single point values. This profit is the difference between the market price of the commodity and the production costs, that depend on land rent, fertilizer costs, water costs, other non-land costs and the crop yield. Additionally, GCAM includes a multi-level nesting structure in order to control the level of substitution between different land types. The land switching is controlled by exogenously defined logit parameters. Yields for each crop are exogenously defined in GCAM. Yields in year 2010 (final calibration year) are based on Food and Agriculture Organization (FAO) and Global Trade Analysis Project (GTAP) data and they are downscaled to all the land subregions. For future yield growth rates, the model uses FAO projections for 2050. For 2100, GCAM assumes that all yields would continue to increase but at decreasing rate. In terms of trade of the commodities produced, this version of the model (GCAM v5.2) has the capability to represent regional agricultural markets. Within each region, imports come from and exports go to a global pool for each single crop, what means that bilateral trade is not modelled. The definition of regional agricultural markets has a significant impact on absolute production quantities and prices of each crop in each region, which are key elements for the marginal damages calculations in this study. All the information related to the agriculture and land use (AGLU) sector can be found in the online documentation^11^ and in Kyle et al. (2011)

In terms of emission module, GCAM tracks the main Greenhouse Gases (GHGs) and air pollutants, namely carbon dioxide (CO_2_), methane (CH_4_), nitrogen dioxide (N_2_O), hydrofluorocarbons (HFCs), black carbon (BC), organic carbon (OC), carbon monoxide (CO), non-methane volatile organic compounds (NMVOC), ammonia (NH_3_), nitrogen oxides (NOx) and sulphur dioxide (SO_2_). The model reports CO_2_ emissions from fossil fuel combustion, industry and transport, and, separately, it also reports land use change emissions. For calibration of emissions in base years, GCAM uses data from the Emissions Database for Global Atmospheric Research (EDGAR) and the Carbon Dioxide Information Analysis Center (CDIAC). For BC and OC calibration, the model uses data from Bond et al. (2004) and Lamarque et al. (2010). Future emission levels will depend on emission factors, activity levels and abatement options. Emission factors are exogenously defined and are different for each region and/or technology, so technology shifts directly affect emission levels. In terms of abatement, the model distinguishes between GHGs and air pollutants. For GHGs, the model incorporates marginal abatement cost curves based on EPA (2013). For air pollutants, GCAM implicitly applies emission controls as a function of per capita GDP (Smith et al. 2005). Therefore, projected emissions of air pollutants would decrease as income increases, even though there is no climate policy explicitly established. Further documentation on the emissions module is available online.^12^

#### TM5-FASST

TM5-Fast Scenario Screening Tool (TM5-FASST) is an air quality source-receptor model developed by the Joint Research Center of the European Commission. The model uses parametrization of meteorological and chemistry data from a full chemistry model (TM5) in order to estimate fine particulate matter (PM_2.5_) and O_3_ concentration levels, and the subsequent impacts on human health or agricultural systems. Detailed documentation of TM5-FASST can be found in Van Dingenen et al. (2018b).

The estimation of O_3_ concentration levels is based on pre-computed source-receptor coefficients (SRCs). These SRCs were estimated by applying single 20% emission perturbations to RCP year 2000 base-run emissions of different O_3_ precursors (NOx and NMVOC^13^) in each region using the TM5 full chemistry model. We note that the model does not include specific perturbation simulations on CH_4_ effect on O_3_. Instead, the CH_4_-O_3_ source-receptor relation is based on results from the first phase of the Hemispheric Transport of Air Pollutants (HTAP1) assessment (Fiore et al. 2008) using the methodology described by Van Dingenen et al. (2009). In summary, concentrations of O_3_, in region *y*, from all the precursors (*i*) emitted in all regions (*x*_*k*_), is calculated as follows (Eq. 6).

(6)
$$C_{O3}(y)=C_{O3,\text{base}}(y)+\sum_{k=1}^{n_{x}}\sum_{i=1}^{n_{i}}SRC_{i,O3}\left[x_{k},y\right]*\left[E_{i}\left(x_{k}\right)-E_{i,\text{base}}\left(x_{k}\right)\right]$$

where *C*_*O*3,*base*_(*y*) = TM5 base-run O_3_ concentration in region *y* (pre-computed, without emission perturbation), *E*_*i*,*base*_(*x*_*k*_) = TM5 base-run precursor *i* emission in region *x*_*k*_ (without emission perturbation), *SRC*_*i*,*O*3_[*x*_*k*_, *y*] = the *i*–to-O3 source-receptor coefficient for source region *x*_*k*_ and receptor region *y,* pre-computed from a 20% emission reduction of component *i* in region *x*_*k*_ relative to the base run, *E*_*i*_(*x*_*k*_) = scenario emission of precursor *i* in region *x*_*k*_.

For the estimation of potential relative yield losses (RYLs) related to O_3_ exposure, the model uses two different region-averaged exposure metrics, which are AOT40^14^ and Mi.^15^ Then, the model calculates RYLs for four different crops (maize, soybeans, rice and wheat) based on exposure–response functions (ERF) taken from Mills et al., (2007) for AOT40 and Wang and Mauzerall, (2004) for Mi. In this study we estimate RYLs by using Mi exposure metric, so, following Wang and Mauzerall (2004), RYL in region *i*, for crop *j* and in period *t* is calculated by the following equation.

(7)
$$RYL_{t,i,j}\left(\text{scen}\right)=1-\frac{\text{exp}{\left(-\frac{M7_{t,i}\left(\text{scen}\right)}{a_{j}}\right)}^{b_{j}}}{\text{exp}{\left(-\frac{25}{a_{j}}\right)}^{b_{j}}}$$

where *a* and *b* are parameters that are defined for each of the four commodities in the mentioned study.^16^

However, in TM5-FASST as in reduced-form models, O_3_ concentrations are calculated using the “steady state” (or immediate) approach, what means that emissions-concentration relation is determined by running a full chemistry model several times using constant parameters (emissions), until an equilibrium concentration for a certain year is reached. Therefore, this approach does not follow the timeline of emissions and do not model the actual response of CH_4_ and O_3_ concentrations, what is named as “transient” approach. Model ensemble experiments in the Hemispheric Transport of Air Pollutants project (HTAP) have demonstrated that the steady state is an adequate approach for measuring the CH_4_-O_3_ relation, without the need for computationally expensive transient computations (Van Dingenen et al. 2018b).

For short-lived O_3_ precursors, the use of the transient or the steady-state approach does not make a difference since the O_3_ concentration is linked to the precursor emission strength on a time scale of days, and SRCs, which average the day-to-day changes on annual basis, reflect adequately the link between annual mean changes in precursors and annual mean changes in O_3_.^17^

However, CH_4_, which is the precursor analyzed in this study, has a half-life of 12 years, so a CH_4_ pulse in a given year will contribute to O_3_ formation over the next 30 years.^18^ Moreover, the scenario analysis presented in this study is based on the effects of different CH_4_ shocks in O_3_-related RYLs in both the shock year and subsequent periods. Therefore, applying the transient approach, what means following the timeline of CH_4_ emissions, is essential for capturing all the potential effects in this study. Consequently, we have adapted the M7 and M12 metrics (Mi) produced by TM5-FASST (steady state) to the transient approach in order to calculate more accurately the O_3_-related marginal damages to crop revenues of different CH_4_ pulses. Our transient *M’i* metric, which in this study is the input for the ERFs that estimate RYLs, is defined as:

(8)
$$M^{\prime }i_{t,i,j}=Mi_{t,i,j}-SS.dO3ppb_{t,i,j}+\text{Trans}.dO3ppb_{t,i,j}$$

where *SS*.*dO*3*ppb*_*t*,*i*,*j*_ is the “steady state” ozone concentration resulting from sustained constant CH_4_ emissions. *Trans*.*dO*3*ppb*_*t*,*i*,*j*_ represents the “transient effect” of CH_4_ emissions on O_3_ formation in a determined year, and it is estimated using regional O_3_ response sensitivities (TM5-FASST SRCs) that are multiplied with a 30-year moving “effective delta CH_4_ emission” which is a weighted sum of annual emissions over all past 30 years, where each year gets a weighting factor depending on the time distance from the considered year. These transient adjustment factors are based on results from the Hemispheric Transport of Air Pollution project, Task 2 (HTAP 2) and Turnock et al., (2018).

## Appendix II: GCAM Crop Categories and Commodity Classification

**Table:**

GCAM region	Countries
Corn	Maize; Maize, Green; Popcorn
FiberCrop	Agave fiber nes; Bastfibres, other; Coir; Cotton lint; Cottonseed; Fiber crops nes; Flax fiber and tow; Hemp tow waste; Jute; Kapok fiber; Manila fiber (abaca); Ramie; Seed cotton; Sisal
FodderGrass	forage Products; Grasses Nes for forage; Rye grass for forage & silage
FodderHerb	Alfalfa for forage and silage; Beets for Fodder; Cabbage for Fodder; Carrots for Fodder; Clover for forage and silage; Green Oilseeds for Silage; Leguminous for Silage; Maize for forage and silage; Pumpkins for Fodder; Sorghum for forage and silage; Swedes for Fodder; Turnips for Fodder; Vegetable Roots Fodder; Vetches
MiscCrop	Apples; Apricots; Areca nuts; Artichokes; Asparagus; Avocados; Bambara beans; Bananas; Beans, dry; Beans, green; Berries nes; Blueberries Brazil nuts with shell; Broad beans, horse beans, dry; Cabbages and other brassicas; Carobs; Carrots and turnips; Cashewapple; Cassava leaves; Cauliflowers and broccoli; Cherries; Cherries sour; Chesnut; Chick peas; Chicory roots; Chilies and peppers, dry; Chilies and peppers green; Cinnamon; Cloves; Cocoa beans; Coffee green; Cow peas dry; Cranberries; Cucumbers and gherkins; Currants; Dates; Eggplants; Figs; Fruit, citrus nes; Fruits, fresh nes; Fruit, pome nes; Fruit, stone nes; Fruit, tropical fresh nes; Garlic; Ginger; Gooseberries; Grapefruit (inc pomelos); Grapes; Hazelnuts with shell; Hops; Kiwi fruit; Kola nuts; Leeks, other alliaceous vegetables; Lemons and limes; Lentils; Lettuce and chicory; Lupins; Mangoes, mangosteens, guavas; Nutmeg, mace and cardamoms; Nuts nes; Okra; Onions dry; Onions, shallots, green; Oranges; Papayas; Peaches and nectarines; Pears; Peas, dry; Peas, green; Pepper (piper spp); Peppermint; Persimmons; Pidgeon peas; Pineapples; Pistachios; Plantains and others; Plums and sloes; Pulses nes; Pumpkin, squash and gourds; Pyrethrum, dries; Quinces; Raspberries; Spices, nes; Spinach; Strawberries; String beans; Tallowtree seed; Tangerines, mandarins, clementines, satsumas; Tea; Tea Nes; Tobacco, unmanufactured; Tomatoes; Vanilla; Vegetables, fresh nes; Vegetables, leguminous nes; Walnuts with shell; Watermelons
OilCrop	Castor oil seed; Groundnuts with shell; Hempseed; Jojoba seed; Kapok fruit; Kapokseed in shell; Karite nuts (sheanuts); Linseed; Melonseed; Mustard seed; Oilseed nes; Olives; Poppy seed; Rapeseed; Safflower seed; Sesame seed; Soybeans; Sunflower seed; Tung nuts
OtherGrain	Barley; Buckwheat; canary seed; Cereals nes; Fonio; Grain, mixed; Millet; Oats; Quinoa; Rye; Sorghum; Triticale
PalmFruit	Coconuts; Oil, palm fruit
Rice	Rice, paddy
RootTuber	Cassava; Potatoes; Roots and tubers, nes; Sweet potatoes; Taro (cocoyam); Yams; Yautia (cocoyam)\
SugarCrop	Sugar beet; Sugar cane; Sugar crops, nes
Wheat	Wheat

## Appendix III: GCAM Regions

**Table:**

GCAM region	Countries
USA	United States
Africa Eastern	Burundi, Comoros, Djibouti, Eritrea, Ethiopia, Kenya, Madagascar, Mauritius, Reunion, Rwanda, Sudan, South Sudan, Somalia, Uganda
Africa Northern	Algeria, Egypt, Western Sahara, Libya, Morocco, Tunisia
Africa Southern	Angola, Botswana, Lesotho, Mozambique, Malawi, Namibia, Swaziland, Tanzania, Zambia, Zimbabwe
Africa Western	Benin, Burkina Faso, Central African Republic, Cote d’Ivoire, Cameroon, Democratic Republic of the Congo, Congo, Cape Verde, Gabon, Ghana, Guinea, Gambia, Guinea-Bissau, Equatorial Guinea, Liberia, Mali, Mauritania, Niger, Nigeria, Senegal, Sierra Leone, Sao Tome and Principe, Chad, Togo
Australia NZ	Australia, New Zealand
Brazil	Brazil
Canada	Canada
Central America and Caribbean	Aruba, Anguilla, Netherlands Antilles, Antigua & Barbuda, Bahamas, Belize, Bermuda, Barbados, Costa Rica, Cuba, Cayman Islands, Dominica, Dominican Republic, Guadeloupe, Grenada, Guatemala, Honduras, Haiti, Jamaica, Saint Kitts and Nevis, Saint Lucia, Montserrat, Martinique, Nicaragua, Panama, El Salvador, Trinidad and Tobago, Saint Vincent and the Grenadines
Central Asia	Armenia, Azerbaijan, Georgia, Kazakhstan, Kyrgyzstan, Mongolia, Tajikistan, Turkmenistan, Uzbekistan
China	China
EU-12	Bulgaria, Cyprus, Czech Republic, Estonia, Hungary, Lithuania, Latvia, Malta, Poland, Romania, Slovakia, Slovenia
EU-15	Andorra, Austria, Belgium, Denmark, Finland, France, Germany, Greece, Greenland, Ireland, Italy, Luxembourg, Monaco, Netherlands, Portugal, Sweden, Spain, United Kingdom
Europe Eastern	Belarus, Moldova, Ukraine
Europe Non EU	Albania, Bosnia and Herzegovina, Croatia, Macedonia, Montenegro, Serbia, Turkey
European Free Trade Association	Iceland, Norway, Switzerland
India	India
Indonesia	Indonesia
Japan	Japan
Mexico	Mexico
Middle East	United Arab Emirates, Bahrain, Iran, Iraq, Israel, Jordan, Kuwait, Lebanon, Oman, Palestine, Qatar, Saudi Arabia, Syria, Yemen
Pakistan	Pakistan
Russia	Russia
South Africa	South Africa
South America Northern	French Guiana, Guyana, Suriname, Venezuela
South America Southern	Bolivia, Chile, Ecuador, Peru, Paraguay, Uruguay
South Asia	Afghanistan, Bangladesh, Bhutan, Sri Lanka, Maldives, Nepal
South Korea	South Korea
Southeast Asia	American Samoa, Brunei Darussalam, Cocos (Keeling) Islands, Cook Islands, Christmas Island, Fiji, Federated States of Micronesia, Guam, Cambodia, Kiribati, Lao Peoples Democratic Republic, Marshall Islands, Myanmar, Northern Mariana Islands, Malaysia, Mayotte, New Caledonia, Norfolk Island, Niue, Nauru, Pacific Islands Trust Territory, Pitcairn Islands, Philippines, Palau, Papua New Guinea, Democratic Peoples Republic of Korea, French Polynesia, Singapore, Solomon Islands, Seychelles, Thailand, Tokelau, Timor Leste, Tonga, Tuvalu, Viet Nam, Vanuatu, Samoa
Taiwan	Taiwan
Argentina	Argentina
Colombia	Colombia

## Appendix IV: Variations in the Seasonal 3-Monthly Mean of 7-h Daytime Ozone in January

The main text shows the changes in the seasonal 3-monthly mean of 7-h daytime ozone (M7) for July, because it is a representative month of the growing season in the Northern Hemisphere, which is the area with the larger crop production. This Appendix shows the variations in O_3_ attribuable to the central CH_4_ pulse for January, which it is a representative month for the growing season in the Southern Hemisphere.

## Appendix V: Comparison Between Transient and Steady-State Approaches

In order to estimate the O_3_ concentration levels, reduced-form models (like TM5-FASST) follow the steady-state approach, which, in contrast to the transient approach, does not consider the timeline of emissions (see Appendix I). In this particular study, it is essential to apply a transient approach, given that methane, which is the O_3_ precursor analyzed in this work, has a half-life of 12 years, so a CH_4_ shock in a certain year would also have an impact on O_3_ concentrations in the subsequent periods. This effect can not be captured with the well-accepted steady-state approach. Therefore, we have adapted our CH_4_-O_3_ relations to a transient approach, as explained in Appendix I. In order to show the implications of using each of these approaches, the following figures analyze the RYLs, using both the transient and the steady state CH_4_-O_3_ relations.

By definition, the effect of the CH_4_ shock on O_3_ concentration following steady-state approach is concentrated on the period where the shock is implemented (*PulseYear*) with no additional effects in subsequent periods. The effect of the shock using the transient approach is based on the 30-year moving “effective delta CH_4_ emission”, with each period getting a weighting factor depending on the distance from the *PulseYear*. Therefore, the transient approach captures not only the damages on the *PulseYear*, but the subsequent impacts related to the atmospheric lifetime of methane. Nevertheless, the impacts on the *PulseYear* will be larger using the steady state approach, as all the effect is concentrated in that period. This is summarized in Fig. 11, which shows the changes in RYLs in USA associated to a CH_4_ shock following both the transient and the steady-state approaches. In terms of total RYLs per period and crop, there is no significant difference between the methods applied, as shown in Figs. 12, 13.

## Appendix VI: Marginal Damages by Region

See Table 2.

**Table 2:** Ozone-related marginal damages to crop revenues per tonne of methane (CH_4_) for the central scenario by region

Region	Marginal damage ($/t-CH_4_)
Africa Eastern	5.7
Africa Northern	14.2
Africa Southern	4.7
Africa Western	10
Argentina	4.8
Australia NZ	0.7
Brazil	27.2
Canada	9.7
Central America and Caribbean	3.1
Central Asia	3.1
China	79.7
Colombia	0.5
EU-12	8.9
EU-15	77.6
Europe Eastern	5.2
Europe Non EU	19.6
European Free Trade Association	0.8
India	43.1
Indonesia	11.7
Japan	4.8
Mexico	10.3
Middle East	17.8
Pakistan	1.7
Russia	5.9
South Africa	2.7
South America Northern	0.6
South America Southern	2.8
South Asia	1.8
South Korea	2.6
Southeast Asia	13.7
USA	98
Total	493

The central scenario represents a 10% increase of projected 2020 USA CH_4_ emissions in 2020 under the SSP2 storyline (Shared Socioeconomic Pathway)

## Appendix VII: Comparison of the Key Climate and Ozone-Related Damages

**Table:**

Study	Description	Damage (2010$/t-CH_4_)
Present study	Agricultural damages from ozone exposure	498
Waldhoff et al (2011)	Climate damages (SC-CH_4_)	1127
Marten and Newbold (2012)		842
Marten et al (2015)		1276
Colbert et al (2020)		1209
Sarofim et al (2017)	Human health damages	1257 (774—1739)
Shindell et al (2017)	Human health, agricultural damages, and ecosystem carbon uptake	3520
